# Workplace Bullying and Its Associated Factors Among Medical Doctors in Residency Training in a Tertiary Health Institution in Plateau State Nigeria

**DOI:** 10.3389/fpubh.2021.812979

**Published:** 2022-01-27

**Authors:** Tolulope O. Afolaranmi, Zuwaira I. Hassan, Benjamin M. Gokir, Abdulrahman Kilani, Raphael Igboke, Kainechukwu G. Ugwu, Chikwendu Amaike, Akinyemi O. D. Ofakunrin

**Affiliations:** ^1^Department of Community Medicine, University of Jos, Jos, Nigeria; ^2^Department of Community Medicine, Jos University Teaching Hospital, Jos, Nigeria; ^3^Faculty of Clinical Sciences, University of Jos, Jos, Nigeria; ^4^Ginza Medical Centre, Jos, Nigeria; ^5^Department of Community Medicine, College of Health and Medical Sciences, Babcock University, Ilishan-Remo, Nigeria; ^6^Department of Pediatrics University of Jos, Jos University Teaching Hospital, Jos, Nigeria

**Keywords:** workplace, bullying, associated factors, medical doctors, residency training, tertiary health institution, Nigeria

## Abstract

**Background:**

Bullying is public health problem globally in workplaces with untold deleterious effects on the health and well-being of individuals at the receiving end. Bullying has been found to disrupt social interaction at workplace thereby creating an unhealthy and seemingly unproductive work environment. Studies have reported varying rates of workplace bullying as high as 83% in Europe, 65% in the Americas and 55% in Asia with very little documented in the contemporary African setting and Nigeria in particular. It therefore became imperative to assess the level of bullying and its associated factors among medical doctors in residency training in a tertiary health institution in Plateau state Nigeria.

**Methodology:**

This was a cross sectional study conducted among resident doctors in Jos University Teaching Hospital between November 2019 and February 2020 using quantitative method of data collection and SPSS version 20 was used for data analysis. Crude and adjusted odds ratios as well as 95% confidence interval were used in this study with a *p*-value of ≤0.05 considered statistically significant.

**Results:**

The mean age of the respondents was 32.3 ± 3.9 years with 78 (62.9%) being 31 years and above. Bullying was currently being experienced by 74 (59.7%) of the respondents with verbal aggression and threats as well as insult and use of derogatory remarks being the forms of bullying experienced by 85.1 and 74.3% of the respondents, respectively. Furthermore, witnessing a colleague being bullied was the sole factor found to be significantly associated with workplace bullying (AOR = 0.18; 95% CI = 0.068–0.449; *p* < 0.001).

**Conclusion:**

Workplace bullying has been found to be in existence and relatively high among medical doctors in residency training in this setting with witnessing someone being bullied as its sole associated factor.

## Introduction

Bullying is a public health problem globally in workplaces with untold deleterious effects on the health and well-being of individuals at the receiving end (1, 2). Workplace bullying has been described as constant and regular negative behaviors toward an employee or its work leading to low sense of dignity self worth (2–4). Bullying has been found to disrupt social interaction at workplace thereby creating an unhealthy and seemingly unproductive work environment (5–7). Studies have reported varying rates of workplace bullying as high as 83% in Europe, 65% in the Americas and 55% in Asia with very little documented in the contemporary African setting and Nigeria in particular (8). It is common place that bullying occurs in all workplace settings of which medical profession is not an exemption. It therefore became imperative to assess the level of bullying and its associated factors among medical doctors in residency training in a tertiary health institution in Plateau state Nigeria. This is opined to bring to light the somewhat submerged and unrecognized levels of workplace bullying in this subset of health care workers and provide the platform for structuring home-grown solutions to mitigating it through identified factors potentiating it.

## Methodology

### Study Setting

This study was conducted in Jos University Teaching Hospital (JUTH), a tertiary health institution founded in 1975 and affiliated with the University of Jos (9). JUTH is a 600-bed capacity facility located in the Lamingo Area of Jos North Local Government Area (LGA) (9). JUTH offers a vast variety of specialized services in the various aspects of healthcare, research and training and serves as a referral center to the surrounding states in the North central, parts of north western and north eastern part of Nigeria. JUTH being a tertiary health facility has the following service delivery units; surgery, internal medicine, obstetrics and gynecology, pediatrics, community medicine, radiology, ophthalmology, pathology, laboratory medicine, otorhinolaryngology, anesthesia, psychiatry and dentistry among others.

### Study Population

The study population comprised of all resident doctors undergoing specialty training in Jos University Teaching Hospital at the time of the study.

### Study Design

A cross-sectional study design conducted between November 2019 and February 2020 to assess the level of workplace bullying, its form and predictors among resident doctors in Jos University Teaching Hospital, Plateau State Nigeria using quantitative method of data collection.

### Sample Size Estimation

The sample size for this study was determined using the appropriate sample size determination formula for a cross sectional study denoted below (10). Where n is the minimum sample size, Z is the standard normal deviate at 95% confidence interval (1.96), q is the complementary probability (1 − p), d is the precision of the study set at 0.05 and p is the prevalence of workplace bullying from previous similar study being 92.0% (1). This gave a sample size of 124 after addition of 10% to cater for non, poor and or incomplete responses.

### Criteria for Inclusion in the Study

All medical doctors in residency training for 6 months and upwards were included in the study while those on either outside posting or leave (annual or sick) were excluded from the study. Six months of training experience was used as the cut off to ensure that the participants had sufficient interaction with both superiors and contemporaries in the course of their respective trainings.

### Sampling Technique

A stratified sampling technique was used in order to ensure representativeness of all the departments of specialty training owing to the fact that these departments had varied number of eligible resident doctors. A list of all the eligible resident doctors from the various departments was obtained, serialized and de-identified with unique departmental codes forming the sampling frame. Following which proportion to size technique was used to obtain the number of participants to be sampled from each of the departments. This was done by dividing the number of resident doctors who had met the inclusion criteria per department (psychiatry—11, surgery and its sub-specialties—54, internal medicine—38, Obstetrics and gynecology—44, pediatrics—23, community medicine—41, ophthalmology—18, otorhinolaryngology—12, hematology—7, family medicine—37 and dentistry—11, radiology—17, medical microbiology—7, chemical pathology—8, histopathology—11, Anesthesia—10) by the cumulative total number of all the eligible resident doctors in all the departments of training (349) multiplied by the sample size of 124 for the study. This gave the following number of resident doctors sampled per department: psychiatry—4, surgery and its sub-specialties—19, internal medicine—14, Obstetrics and gynecology—15, pediatrics—8, community medicine—14, ophthalmology—6, otorhinolaryngology—4, hematology—3, family medicine—13 dentistry—4, radiology—6, medical microbiology—3, chemical pathology—3, histopathology—4, anesthesia—4). Thereafter, the respective departmental list was drawn and numbers were allocated to the all the eligible respondents in ascending order forming the departmental sampling frame from which computer generated table of random numbers was used to select determined number of resident doctors for each department, respectively, without replacement.

### Data Collection Instrument

A semi-structured self-administered questionnaire adapted from a previous studies comprising of three sections; socio-demographic characteristics, prevalence of workplace bullying and pattern of workplace bullying (11, 12). Three research assistants were trained on the content, method of administration and retrieval of filled questionnaire prior to the commencement of the study by the principal researcher. Cronbach alpha reliability assessment of the questionnaire was done using SPSS software version 20 where an overall Cronbach alpha score of 0.81 was obtained. The data collection instrument was pretested in among resident doctors in another training institution among 10% of the calculated minimum sample size. This was done in order to correct any ambiguity in the questionnaire and also to estimation of time of administration of the questionnaires.

### Data Collection

Data were collected using a paper-based semi-structured self-administered questionnaire. The eligible and selected participants identified with the help of the various departmental focal persons were sampled in their respective departments daily. The departmental focal person helped in the retrieval of all the filled questionnaires for onward collection by the research assistants. Upon the receipt of all the filled questionnaires from departmental focal persons, the trained research assistant reviewed all the questionnaires for completeness and appropriateness of the responses as required. The questionnaires not completely filled were returned for proper filling and retrieved back. Confidentiality and anonymity of the information provided by the participants were assured and maintained.

### Ethical Consideration

Ethical clearance was obtained from Jos University Teaching Hospital Institutional Human Research Ethical Committee (JUTH/DCS/IREC/127/XXX/2137). Written informed consent was obtained from all the respondents with confidentiality and anonymity of their responses assured and maintained.

## Grading of Response

Explanatory variables in this study were categorized as demographic characteristics of the respondents. The demographic characteristics included age which was age as at last birthday and then categorized into ≤ 30 and 31 years and above after plotting a percentile graph and 30 years was found to bifurcated the data into two halves. Furthermore, sex of the respondents was obtained and categorized as male or female based on the responses obtained. Other explanatory variables assessed were marital status assessed as single or married, level of training graded as registrar and senior registrar. Senior registrars are those doctors in residency training who had passed the part—one fellowship/college examinations after spending the mandatory 3 years junior residency phase and are the final phase of their specialty training. Additionally, information on duration in specialty training was elicited in years and graded as ≤3 and 4 years and above. Three years was used as the cut-off being the mandatory period for junior residency phase. The outcome measure of the study was the experience of any act of bullying which was elicited with a yes or no response to the question “Have you experienced any form of workplace bullying in the last 6 months in the course of your training?” Following which the respondents were asked to indicate the acts of bullying they had experienced. Bullying was adjudged to have been experienced if the respondents had been exposed to any acts of intimidation, humiliation, degrading, misuse or abuse of power, authority or position which caused any feelings of defenselessness as well as undermining his/her sense of dignity while at work within the last 6 month (3, 4).

## Data Analysis

Data analysis was carried out using Statistical Package for Social Sciences (SPSS) version 20. Descriptive statistical analysis was carried out on quantitative variables such age of the respondent using mean and standard deviation as the summary indices upon the fulfillment of the assumptions of normality. Other explanatory variables such as age group, sex, marital status, religion, level of training, duration of training and field of specialization were presented using frequency table expressed in frequencies and percentages. The primary outcome variable was workplace bullying expressed as experienced and not experienced presented in a frequency table. A stepwise model approach to logistic regression was used in determining the factors influencing workplace bullying where each of the explanatory variables was fed into the logistic regression model singly following which crude odds ratios and 95% confidence intervals were generated, respectively. Furthermore, all these factors were then fed cumulatively into the logistic regression model to establish interaction and allow for these factors to adjust for one another. Adjusted odds ratio and 95% confidence interval were used as point and interval estimates of the measure of the effects of these factors on the experience of workplace bullying. Additionally, a probability value of <0.05 was considered statistically significant.

## Results

The mean age of the respondents was 32.3 ± 3.9 years with 78 (62.9%) being 31 years and above. With regards to sex distribution of the study participants, 91 (73.4%) were males while 95 (76.6%) were in the registrar cadre. Surgery and its sub-specialties accounted for 19 (15.3%) of the respondents, while Internal Medicine, Obstetrics and Gynecology and Community Medicine accounted for 14 (11.3%), 15 (12.1%), and 14 (11.3%) of the respondents, respectively (Table 1).

**Table 1:** Socio-demographic characteristics of the respondents.

**Characteristics**	**Frequency (*n* =124)**	**Percentage**
**Age (Years)**		
≤30	46	37.1
31 and above	78	62.9
	Mean ± SD	
Mean age	32.3 ± 3.9 years	
**Sex**		
Male	91	73.4
Female	33	26.6
**Religion**		
Christianity	120	96.8
Islam	4	3.2
**Level of training**		
Registrar	95	76.6
Senior Registrar	29	23.4
**Duration in specialty training (years)**		
≤3	91	73.4
4 and above	33	26.6
**Marital Status**		
Single	62	50.0
Married	62	50.0
**Field of specialization**		
Surgery and its sub-specialties	19	15.3
Internal Medicine	14	11.3
Obstetrics and Gynecology	15	12.1
Peadiatrics	8	6.5
Family Medicine	13	10.5
Community Medicine	14	11.3
Laboratory Medicine	13	10.5
Others*	28	22.6

SD, Standard Deviation. *Psychiatry, radiology ophthalmology, otorhinolaryngology, anesthesia, dentistry.

*Laboratory Medicine, Hematology, medical microbiology, chemical pathology, histopathology*.

Bullying was currently being experienced in the course residency training at one point or the other by 74 (59.7%) of the respondents while 86 (69.4%) stated that they had witnessed at least a colleague being bullied since commencement of residency training. Verbal aggression and threats, insult and use of derogatory remarks as well as intimidation by superiors were the forms of bullying experienced by 85.1, 74.3, and 60.8% of the respondents, respectively, among participants who had experienced bullying (Table 2).

**Table 2:** Prevalence and forms of workplace bullying.

**Variables**	**Frequency *n* = 124**	**Percentage**
**Witnessed colleague(s) being bullied**		
Yes	86	69.4
No	38	30.6
**Current experience of bullying**		
Experienced	74	59.7
Not experienced	50	40.3.
**Direction of bullying experienced (*****n*** **=** **74)**		
Superior to subordinate	74	100.0
Among peers	0	0.0
Forms of bullying*(***n*** **=74)**		
Verbal aggregation and threats	63	85.1
Playing of mind games	28	37.8
Social isolation	20	27.0
Insults or derogatory remarks	55	74.3
Intimidation	45	60.8
Neglecting one's opinion	38	51.4

Furthermore, witnessing a colleague being bullied was the only factor influencing bullying in the workplace in this study. The odds of being bullied for those who had not witnessed any of their colleagues being bullied was 0.18 times those who had witnessed same (95% CI = 0.068–0.449; *p* < 0.001) (Table 3).

**Table 3:** Multiple logistic regression of factors influencing workplace bullying.

**Factors**	**COR (95%CI)**	***P*-value**	**AOR (95% CI)**	***P*-value**
**Age group (years)**				
≤30	1.42 (0.68–2.97)	0.354	1.16 (0.43–3.12)	0.763
31 and above	1	–	1	–
**Sex**				
Female	0.45 (0.20–1.02)	0.054	0.84 (0.29–2.41)	0.747
Male	1	–	1	–
**Marital status**				
Single	1.30 (0.64–2.69)	0.464	1.64 (0.63–4.28)	0.312
Married	1	–	–	–
**Level of training**				
Senior Registrar	0.59 (0.25–1.44)	0.247	0.75 (0.20–2.82)	0.667
Registrar	1	–	–	–
**Witnessing a colleague being bullied**				
No	0.165 (0.07–0.38)	<0.001	0.18 (0.07–0.45)	<0.001
Yes	1	–	–	–
**Duration of training (years)**				
4 years and above	0.89 (0.40–1.99)	0.774	0.42 (0.12–1.49)	0.180
≤3 years	1	–	–	–
**Field of specialization**				
Surgery and its sub-specialties	2.20 (0.64–7.58)	0.213	1.73 (0.43–6.96)	0.442
Internal medicine	4.67 (0.40–53.93)	0.217	3.19 (0.24–43.22)	0.384
Obstetrics and gynecology	1.37 (0.29–6.36)	0.695	1.08 (0.20–5.91)	0.458
Peadiatrics	1.05 (0.26–4.32)	0.946	0.87 (0.17–4.47)	0.867
Family medicine	0.47 (0.07–3.34)	0.448	0.41 (0.05–3.76)	0.433
Community medicine	10.50 (1.02–18.58)	0.002	5.21 (0.24–6.94)	0.055
Laboratory medicine	1.94 (0.32–11.76)	0.469	1.23 (0.15–9.88)	0.847
Others^*^	1	–	–	–

* *Psychiatry, radiology ophthalmology, otorhinolaryngology, anesthesia, dentistry*.

*Laboratory Medicine, Hematology, medical microbiology, chemical pathology, histopathology; COR, Crude Odds Ratio; AOR, Adjusted Odds Ratio*.

*Age group, sex, marital status, level of training, witnessing colleague being bullied, duration of training and field of specialization were all adjusted for in the multiple logistic regression model*.

## Discussion

Workplace bullying is any anti-social behavior in the workplace resulting in distress, discomfort, physical or psychological harm (13). The prevalence of workplace bullying in this study was relatively high with slightly above half of the respondents having experienced bullying at one point or the other in the course of their postgraduate specialization training. This is similar to what was reported in a Greek study though conducted among health care providers in just one unit of the hospital as against this study having doctors in training from all the different fields of specialty (6). Furthermore, varying burdens of workplace bullying were also reported in studies conducted in different climes with lower prevalence rate found in those carried out in Spain, Taiwan, Portugal and Cyprus in comparison to findings of this study while higher rates were recorded in studies conducted in Turkey, Nigeria and Egypt (5, 14–19).

This variation could be attributable to the difference in the cadres of health care workers studied, the methods of assessment of workplace bullying as well as the culture and societal perception of bullying in these different study settings. Importantly, a harmonious work environment is tantamount to having good treatment outcomes for the end service users, motivation and improved productivity as well as job satisfaction for the health providers and in this context, a fulfilling residency training experience for the trainees. This implies that workplace bullying should either be non-existent or reduced to the barest minimum if the health care system will achieve its intended purposes and produce specialists who would value the importance of good interpersonal relationship and peaceful co-existence.

Furthermore, over two-third of the respondents had witnessed at least a colleague being bullied within a period of 6 months prior to the study which is consistent with what was reported in another study conducted in Europe (6). This has brought to light that bullying cuts across geographic locations and regions and if it goes on unchecked, the possibility of it being taken as the norm and modeled as part of daily routine is high. Hence, it may be imperative for health institutions to develop or have in place anti–workplace bullying system and reporting channels as measures of curbing it. Furthermore, verbal aggression and threats, intimidation, social isolation as well as neglect of one's opinion were expressed as the forms of bullying experienced in this study which corroborate the findings of other studies in addition to direct hostile behavior, being continuously interrupted, being gossiped about, failure to respect privacy, excessive workload, degrading remarks, physical abuse with rage and anger among others being reported (5, 13, 15–19). This is a pointer to the fact that these anti-social behaviors exist in workplaces globally with shared similarities in different countries and regions making workplace bully an emerging public health problem requiring urgent global attention.

With regards to the factors influencing workplace bullying among doctors in the residency training, witnessing a colleague being bullying was found to be the sole factor influencing bullying. However, other similar studies have reported factors such as gender, age, number of years in service, workload, level of education and shift employment as factors influencing bullying in the work place (6, 14, 17, 18). It however, imperative to state that observing someone being bullied could subtly over a period of time model individuals to seeing bullying as normal and then potentiating the desire for bullying other in such individual. Additionally, in view of the diversity of predictors of workplace bullying among health care worker, it important to be context and settings specific in providing interventions targeted at addressing this dangerous act so as to achieve meaningful results. The study stands to contribute to the existing body of knowledge on the existence of bullying among health care providers cutting across various specialties. This in itself has brought to light that bullying is not restricted to any profession or career pathway. However, assessment of bullying in this study was self reported and limited to only medical doctors in specialty training which in a way could be a limitation to the generalizabilty of the findings of the study. Additionally this study had used only quantitative method of data collection while the use of mixed method approach may have provided more insight into more hidden factors influencing bullying in the group of respondents.

## Conclusion

Workplace bullying has been found to be in existence and relatively high among medical doctors in residency training in this setting with witnessing someone being bullied as its sole predictor. Hence, it is imperative that the acts of bullying be discouraged through sensitization and education so as to forestall modeling bullying as a integral part of specialty training.

## Data Availability Statement

The raw data supporting the conclusions of this article will be made available by the authors, without undue reservation.

## Ethics Statement

The studies involving human participants were reviewed and approved by Institutional Research Ethics Committee of Jos University Teaching Hospital. The patients/participants provided their written informed consent to participate in this study.

## Author Contributions

TA and ZH participated in conceptualization and design of the study, literature review, analysis and interpretation of results, drafting and revising the manuscript, and final approval prior to submission for publication. BG, AK, RI, KU, CA, and AO participated in the design of the study, literature review, analysis and interpretation of results, drafting and revising the manuscript, and final approval prior to submission for publication. All authors agreed to be accountable for the content of the work.

## Funding

This study was supported by the Fogarty International Center (FIC), Office of the Director (OD/NIH), National Institute of Neurological Disorders and Stroke (NINDS/NIH), and the National Institute of Nursing Research (NINR/NIH) of the National Institutes of Health under Award Number D43 TW010130.

## Author Disclaimer

The content is solely the responsibility of the authors and does not necessarily represent the views of the National Institutes of Health.

## Conflict of Interest

The authors declare that the research was conducted in the absence of any commercial or financial relationships that could be construed as a potential conflict of interest.

## Publisher's Note

All claims expressed in this article are solely those of the authors and do not necessarily represent those of their affiliated organizations, or those of the publisher, the editors and the reviewers. Any product that may be evaluated in this article, or claim that may be made by its manufacturer, is not guaranteed or endorsed by the publisher.
