# Supplementary material for: Workplace Bullying and Its Associated Factors Among Medical Doctors in Residency Training in a Tertiary Health Institution in Plateau State Nigeria
Source: Front Public Health. 2022 Jan 27;9:812979. doi: 10.3389/fpubh.2021.812979 (PMC8830776; doi:10.3389/fpubh.2021.812979)
Supplement: Supplementary file 1 [file Data_Sheet_1.PDF]

QUESTIONNAIRE ON THE ASSESSMENT OF THE PREVALENCE OF WORKPLACE BULLYING AMONG RESIDENT DOCTORS IN JOS UNIVERSITY TEACHING HOSPITAL

SECTION A: DEMOGRAPHIC CHARACTERISTICS

Instructions: Please kindly fill in the Blank and thick as appropriate.

Initials: ……………………………………………………………

1. Age: ………………………………………………………………..
2. Religion: …………………………………………………………

4. Sex: ( ) Male ( ) Female

5. Marital Status: ………………………………………………….

6. Department: …………………………………………………….

7. Years in Specialty training: …………………………….

8. Level of training: ( ) Registrar ( ) Senior registrar

SECTION B: PREVALENCE OF BULLYING

9. Have you heard of workplace bullying? (a) Yes (b) No

10. If yes what is workplace bullying..

11. What are the forms of bullying in the workplace that you know?

………………………………………………………………

………………………………………………………………

………………………………………………………………

………………………………………….........................

………………………………………………………………

12. Have you experienced any form of workplace bullying in the last 6 months in the course of your training? Yes ( ) No ( )

13. If Yes, kindly indicate

Verbal aggression and threats ( )

Playing of mind games ( )

Social isolation ( )

Insulting remarks or gossip ( )

Physical violence ( )

Intimidating acts ( )

Neglect of opinion ( )

Others, Kindly Specify……………………………

14. How often did it occur within the last 6 months?

a) Once ( )
b) 2 - 5 times ( )
c) Repeatedly ( )

15. What was the direction of the bullying?

Superior to Subordinate ( )

Subordinate to Superior ( )

Among peers or contemporaries ( )

16. Which category of doctors in training is most likely to be victims of bullying? (Thick appropriately)

a) Junior Residents ( )
b) Senior Residents ( )
c) Consultants ( )
d) Others please specify ………………………………………………………….

17. Have you ever witnessed anyone being bullied in the course of your specialty training?

Yes ( ) No ( )

18. If yes, kindly indicate the form below

Verbal aggression and threats ( )

Playing of mind games ( )

Social isolation ( )

Insulting remarks or gossip ( )

Physical violence ( )

Intimidating acts ( )

Neglect of opinion ( )

Others, kindly specify……………………………

19. What was the direction of the bullying?

Superior to Subordinate ( )

Subordinate to Superior ( )

Among peers or contemporaries ( )

Thank you
